# Alterations in center-surround contrast suppression in patients with major depressive disorder

**DOI:** 10.1038/s41598-024-78584-z

**Published:** 2024-11-15

**Authors:** Kathrin Nickel, Sven P. Heinrich, Malina Beringer, Dominique Endres, Kimon Runge, Sebastian Küchlin, Simon Maier, Michael Bach, Katharina Domschke, Ludger Tebartz van Elst, Evelyn B. N. Friedel

**Affiliations:** 1 Department of Psychiatry and Psychotherapy, Medical Center – University of Freiburg, Faculty of Medicine, University of Freiburg, Freiburg, Germany; 2 Eye Center, Medical Center - University of Freiburg, Faculty of Medicine, University of Freiburg, Freiburg, Germany; 3 German Center for Mental Health (DZPG), Partner Site Berlin, Berlin, Germany; 4 Faculty of Biology, University of Freiburg, Freiburg, Germany

**Keywords:** Depression, Contrast suppression, Center-surround contrast suppression, Contrast sensitivity, Retina, Depression, Human behaviour

## Abstract

Previous pattern electroretinogram studies indicate reduced retinal contrast gain in patients with a major depressive disorder (MDD) which may contribute to alterations in visual perception. In line, psychophysical investigations reported elevated contrast thresholds in MDD. This study aims to gain insights into higher-level processing of visual information in MDD by evaluating contrast suppression. We examined contrast suppression of 21 MDD patients and 23 healthy controls (HC) using four different stimulus conditions (spatial frequencies 6.3 and 12.6 cpd at 30% and 60% background contrast) in a psychophysical test. Participants were instructed to adjust perceived contrasts between two vertical target patches, embedded in a horizontally or vertically oriented grid background. With finer stimulus gratings, MDD patients exhibited less contrast suppression compared to HC, particularly when the stimulus contrast was high (*p* = 0.006; MDD vs. HC =  − 45%). Contrast suppression in the HC group was significantly reduced for the coarse compared to the fine grating, while contrast suppression scores in MDD did not change with the spatial properties of the stimulus. The reduced contrast suppression in patients with MDD supports the hypothesis of altered dopaminergic neurotransmission and could be attributed to alterations in the retinal receptive fields or in dysfunctional adaptation mechanisms in depression.

## Introduction

Previous studies reported altered visual processing in patients with a major depressive disorder (MDD) and detected reduced retinal contrast gain assessed with the pattern electroretinogram (PERG) which normalizes following remission^1,2^. The reduction in retinal contrast gain points towards a possible diminished contrast signal from the retina to the cortex which might result in an altered perception of visual stimuli^3^. In line, in psychophysical investigations, elevated contrast discrimination thresholds^4^ and contrast thresholds^5^ were found in unmedicated and medicated patients with MDD, similarly to earlier observations in patients with Parkinson’s disease^6–9^. This supports the hypothesis that altered dopaminergic neurotransmission might play a key role in the pathophysiology of depression and alterations in visual contrast perception in MDD^4^.

Center-surround contrast suppression typically occurs when a luminance modulated central patch (e.g. sinusoidal grating) of low contrast is presented on a high contrast background pattern with similar spatial features like the same orientation (collinear)^10,11^ and spatial frequency^12,13^. Contrast suppression is less pronounced when the center and surround patterns differ in orientation, particularly evident when they are orthogonal^11^. This orientation specificity suggests involvement of post-retinal processing sites, such as the lateral geniculate nucleus^14^ and cortical areas^3,15^, in contrast suppression.

Brightness induction, on the other hand, is thought to originate on the retinal level^16^. For instance, two test patches of identical physical luminance appear different in brightness when embedded in a luminance gradient^16^, most likely due to lateral inhibition between the surrounding and both center patches.

So far, one pioneering study has investigated contrast suppression in MDD patients with a psychophysical test. Salmela et al.^3^ examined 111 patients with unipolar depression, bipolar disorder and borderline personality disorder with baseline major depressive episodes compared to 29 healthy controls (HC) with a contrast suppression test to assess cortical processing by examining the perceived contrast of gratings presented with either collinear or orthogonally oriented backgrounds. They detected significantly lower contrast suppression in MDD while no difference in brightness induction was discerned. It was suggested that diminished contrast suppression might be attributed to reduced retinal feedforward or cortical feedback signals^3^.

### Aims of the study

The aim of the study was to gain further insights into the visual contrast perception in patients with MDD. For this purpose, we evaluated center-surround contrast suppression in MDD and HC using different stimulus conditions (varying spatial frequency and overall contrasts) in a psychophysical test. Based on the results described by Salmela et al.^3^, we assumed reduced contrast suppression in MDD patients compared to HC.

## Methods

### Participants

The Ethics Committee of the University of Freiburg approved the study (Approval ID: 314/18). The study was conducted in accordance with the guidelines of the Declaration of Helsinki after obtaining written informed consent from all participants.

We recruited 27 patients with a severe depressive episode from the outpatient clinic of the Department of Psychiatry and Psychotherapy of the University Medical Center Freiburg and 26 age- and sex-matched HC. Study participants aged between 18 and 65 years were included. The diagnosis was established by an experienced senior psychiatrist according to the criteria of the International Classification of Diseases, 10th revision (ICD-10). Patients diagnosed with a severe depressive episode (ICD-10: F32.2) or a recurrent depressive disorder, current episode severe (ICD-10: F33.2) were enrolled in the study. Psychotic symptoms, substance abuse or bipolar disorder were exclusion criteria. Moreover, the Montgomery-Åsberg Depression Rating Scale (MADRS)^17^ was collected by a senior specialist in psychiatry and psychotherapy. Patients and HC additionally filled in the following ratings and questionnaires: the Beck Depression Inventory (BDI-II)^18^, a self-assessment questionnaire for depressive symptoms, the Autism-Spectrum Quotient (AQ) and the Empathy Quotient (EQ)^20^ to rule out autistic symptoms and the Wender Utah Rating Scale (WURS-k)^21^ to evaluate symptoms of an attention-/deficit-hyperactivity disorder in childhood. Moreover, the Structured Clinical Interview for DSM (SCID-I and -II^22^) and the Symptom Checklist (SCL-90-R^23^) were gathered to assess psychiatric diseases and rule them out in the HC group. In addition, the Fagerström Test for Nicotine Dependence (FTND^25^) and an anamnesis of coffein and alcohol consumption was collected from all participants.

For the HC group, the presence of a mental disorder was defined as an exclusion criterion. For demographic and clinical characteristics please see Table 1.

**Table 1:** Demographic and psychometric data.

Parameter	MDD (N = 21)	HC (N = 23)	*p*-value
ICD-10 diagnosis: F32.2/F33.2	12 (57%)/9 (43%)	‒	‒
Sex: female/male	17 (81%)/4 (19%)	18 (78%)/5 (22%)	0.562 (ns)#
Age in years	29 (21,36); 19‒61	32 (23,36); 21‒61	0.651 (ns)#
Visual acuity as logMAR	− 0.08 (− 0.17, − 0.03); − 0.22‒0.09	− 0.14 (− 0.19, − 0.10); − 0.29‒0.05	0.125 (ns)#
Antidepressant medication yes/no	13 (62%)/8 (38%)	‒	‒
Days of antidepressant medication	7 (4,11); 1‒14	‒	‒
Antidepressant medication:		‒	‒
SSRI	1 (5%)	‒	‒
SNRI	2 (9%)	‒	‒
Mirtazapine	4 (18%)	‒	‒
SSRI + Mirtazapine	4 (18%)	‒	‒
SNRI + Mirtazapine	2 (9%)	‒	‒
MADRS	36^+^ (35, 40); 28‒45	‒	‒
BDI-II	29 (25, 40); 14‒53	2^+^ (1, 6); 0‒11	< 0.001 (*)#
AQ	20 (16, 22); 9‒28	22^+^ (19, 25); 16‒27	0.219 (ns)#
EQ	44 (41, 49); 29‒52	47^+^ (45, 49); 38‒59	0.070 (ns)#
WURS-k	8 (3, 13); 0‒42	5^+^ (2, 11); 0‒30	0.522 (ns)#
Smoking: yes/no	3 (14%)/18 (86%)	4 (17%)/19 (83%)	0.627(ns)#
FTND	N = 3: 3 (2, 4); 2‒4	N = 4: 2 (1, 2); 1‒3	0.403 (ns)#
Coffein intake	2 (1, 3); 0‒3	3 (1, 3); 0‒3	0.561 (ns)#
Alcohol intake	1 (1, 2); 0‒3	2 (1, 2); 1‒2	0.200 (ns)#

Numerical data are summarized by the median (1st and 3rd quartiles) and the range (min‒max), categorial data are depicted as the number of observations. Categorical data from both groups were compared using (*X*^2^) testing for independence. The differences in medians between MDD and HC were used for numerical data. Statistical comparisons and *p*-value computation were all based on permutation tests (10,000 replicates).

*MDD* major depressive disorder, *HC* healthy controls, *N* number of participants, *ICD-10* International Statistical Classification of Diseases and Related Health Problems version 10, *F32.2* severe depressive episode without psychotic symptoms (ICD-10), *F33.2* recurrent depressive disorder, current episode severe without psychotic symptoms (ICD-10), *ns* not significant, # no FDR adjustment, *SNRI* serotonin and norepinephrine reuptake inhibitor (venlafaxine), *MADRS* Montgomery-Åsberg Depression Rating Scale^17^, ^+^1 missing data set, *BDI-II* Beck Depression Inventory II^18^, *Significant, *AQ* Autism Spectrum Quotient^19^, *EQ* Empathy Quotient^20^, *WURS-k* Wender Utah Rating Scale^21^, *FTND* Fagerström Test for Nicotine Dependence^24^; coffein and alcohol intake was rated as: never = 0, rarely = 1, occasionally = 2, daily = 3.

For all study participants, ophthalmological diseases (apart from correctable refractive errors), myopia greater than −6 D or hyperopia greater than +6 D, or a monocular decimal visual acuity worse than 0.8 decimal (0.1 logMAR) in the Freiburg Visual Acuity and Contrast Test (FrACT)^25^, the presence of somatic diseases such as diabetes mellitus, arterial hypertension or seizures were further exclusion criteria. All participants were examined with optical coherence tomography to screen for the presence of incidental ophthalmological findings by a specialist in ophthalmology.

### Contrast suppression test

**Fig. 1:**
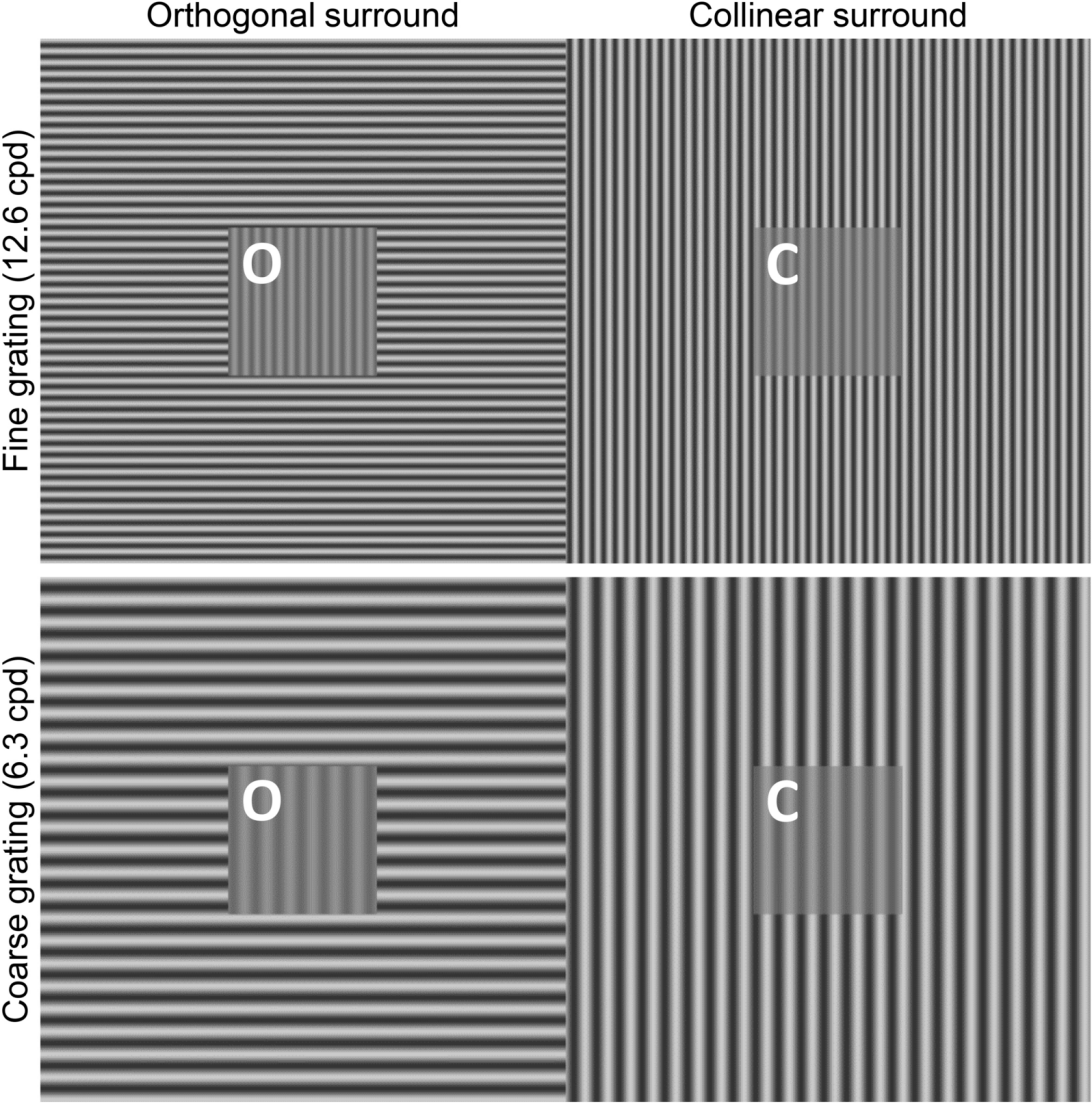
Contrast suppression tests. Schematic representation of gratings used to test contrast suppression. Upper row: fine grating with a spatial frequency of 12.6 cpd; lower row: coarser grating with a spatial frequency of 6.3 cpd. Gratings depicted here have 60% background and 20% target contrast (conditions with 30% background contrast are not shown). Centrally located test patches were vertical gratings presented on either orthogonal (O: left patch) or collinear background (O: right patch). Both test patches have identical contrast (20%), due to contrast suppression effects when background and target have same orientation, the contrast of test patch C (collinear condition) is perceived to be reduced. The letters O and C were not present in the actual stimuli.

As shown in Fig. 1, two vertically oriented target grid patches were presented on either an orthogonally (Fig. 1, left-hand (O)) or a vertically oriented background grating (Fig. 1, right-hand (C)) of the same spatial frequency, but of lower contrast.

Because contrast suppression is specific to orientation and typically occurs when target and background have the same orientation (collinear)^11^, the perceived contrast of test patch C is reduced, compared to test patch O (Fig. 1).

We tested two different spatial frequencies and background contrasts of patterns: a fine (12.6 cycles per degree (cpd)) and a coarser grating (6.3 cpd). For both spatial frequencies, the background pattern was either high (60% Michelson contrast) or low (30% Michelson contrast) in contrast. Test patches had always two-third lower contrast compared to the background grating, e.g. with 60% background contrast, both test patches would have 20% contrast when equally adjusted (same contrast of patch O and C in Fig. 1), while test patches on 30% background contrast would have 10% contrast each when equally adjusted.

Participants were provided with a keypad and instructed to align the contrasts of the centrally located target patches to appear identically by pressing arrow keys up (increase contrast in O and decrease contrast C) or down (increase contrast in C and decrease contrast O) and stop if the point of subjective similarity was achieved. At this point, contrasts of both test patches (O and C) were saved for further analysis. Participants repeated this procedure twice for all conditions.

Based on this stimulus paradigm, the boundaries of target contrasts could reach 0% for O and 40% for C in the condition with 60% background contrast, and 0% for O and 20% for C in the condition with 30% background contrast (“maximum contrast suppression”), or vice versa.

The test applied to measure contrast suppression can be viewed at the following link: https://michaelbach.de/ot/lum-contrastSuppr/capp/ (retrieval date: 13th March, 2024).

Stimuli were presented at 180 cm distance on a laptop screen (14" LED-backlit display, 1920 × 1080 pixels resolution) under normal room lighting conditions. The stimulus field (encompassing both quadratic background gratings) covered an area of 7.63° × 3.82° of visual angle (24 × 12 cm). Both centrally located target test patches covered a visual angle of 1.11° × 1.11° (3.5 × 3.5 cm) each.

### Data preparation and statistical analysis

With “R” in Rstudio^26^ and the “tidyverse” core packages^27^, data were prepared for further analysis, statistically analyzed and depicted graphically.

After data exclusion due to ophthalmological findings or low visual acuity, outliers in the contrast suppression data were identified (values beyond 1.5 × interquartile range, considering all participants) and precluded from further analysis. Bland–Altman analyses^28–30^ of limits of agreement (LOA) were calculated for both groups and all conditions for participants providing data from both repetitions (Supplementary Fig. 1). Data of both repetitions were averaged for all participants. Similar to Salmela et al.^3^, contrast suppression was calculated as the difference in contrast from the collinear and the contrast in the orthogonal patches in relation to the total contrast of both test patches ([C−O]/[C+O]).

We additionally computed the relative contrasts of both test patches by dividing the individual target contrasts of patch O and C by the corresponding target contrasts for matching conditions (e.g. 60% background contrast and 20% target contrast: contrast of O and C were divided by 20%) for graphical representation and to assess if relative contrasts of patches O and C differ in all stimulus conditions.

All statistical analyses and *p*-value computations were based on non-parametric permutation tests (10,000 replicates; “infer” package^31^). Differences in medians (e.g. MDD vs. HC) were used for numerical data, Chi-squared coefficients (*X*^2^) for categorial demographic data, assessing independence. Spearman correlation coefficients (*rho*) were computed using the “correlation” package^32^ and the “jmuOutlier” package^33^ for permutation testing and *p*-value calculation. Significance level was defined as α = 0.05 and adjusted using a false-discovery-rate (FDR) controlling procedure^34^ considering the four conditions of the contrast suppression test. Analysis of demographic and psychometric as well as correlation data were performed without FDR adjustment.

For *p*-value computations, we assumed (based on previous literature) contrast suppression in the MDD group to be less pronounced compared to HC. No assumptions were made for other comparisons.

To ensure comparability with other studies, we also provide *p*-values of parametric statistics (t-tests), Cohen’s *d* and Pearson’s correlation coefficients (*r*). Significance levels were not adjusted for the additional parametric analysis.

## Results

### Participants

Table 1 depicts demographic and psychometric data of study participants. We recruited 27 MDD patients, of whom two had to be excluded due to ophthalmological findings (suspicion of dome-shaped macula in both eyes; perifoveal pigment epithelial alterations in one eye), one due to low visual acuity in one eye (0.5 decimal/0.3 logMAR), two had to be excluded because of their psychiatric medication (bupropion and trimipramine) and one aborted the examination.

Of the remaining 21 patients (17 female, 4 male), 12 were diagnosed with a severe depressive episode (ICD-10: F32.2) and 9 with a recurrent depressive disorder, current episode severe (ICD-10: F33.2). Eight MDD patients were medication-naïve, 13 had received antidepressant medication (see Table 1), whereby medication intake did not exceed 14 days. An experienced specialist in psychiatry and psychotherapy ensured that no remission of depressive symptoms had occurred at the time of the participation.

Out of the 26 initially recruited HC, two had to be excluded due to ophthalmological findings (suspicion of pigment epithelial alteration with associated photoreceptor atrophy), one due to an elevated BDI-II score. Finally, 23 HC (18 female, 5 male) could be included in the analysis.

Patients with MDD exhibited significantly higher depressive symptoms according to their BDI-II scores compared to HC (see Table 1).

An overlapping study cohort was also examined with the contrast test branch of the FrACT^25^ (using Landolt-Cs) to assess contrast sensitivity, with the PERG and also with the flash electroretinogram (fERG), to evaluate retinal ganglion cell responses and other components of the fERG in patients with MDD^50^.

### Contrast suppression

Prior to averaging data from repetitions per individual and condition, detected outliers were removed. Overall, five MDD and two HC showed outliers. For the 12.6 cpd and BGC 60% condition, data of one patient with MDD showed outliers in both repetitions, two MDD in only one. No outliers were detected in the 6.3 cpd and BGC 60% condition. Both repetitions of one MDD and single repetition of two MDD and one HC had to be removed in the 12.6 cpd and BGC 30% condition. In condition 6.3 cpd and BGC 30%, data of one MDD and one repetition of one HC had to be further removed as outliers (see Supplementary Fig. 1 for comparison).

When gratings with high background contrast (BGC 60%) and spatial frequency (12.6 cpd) were presented, contrast suppression was significantly reduced in patients with MDD compared to HC (*p* = 0.006, MDD vs. HC: −45%) while contrast suppression in MDD rather resembles HC level using the fine grating (12.6 cpd) with lower BGC (30%) (*p* = 0.209, MDD vs. HC: −16%).

**Fig. 2:**
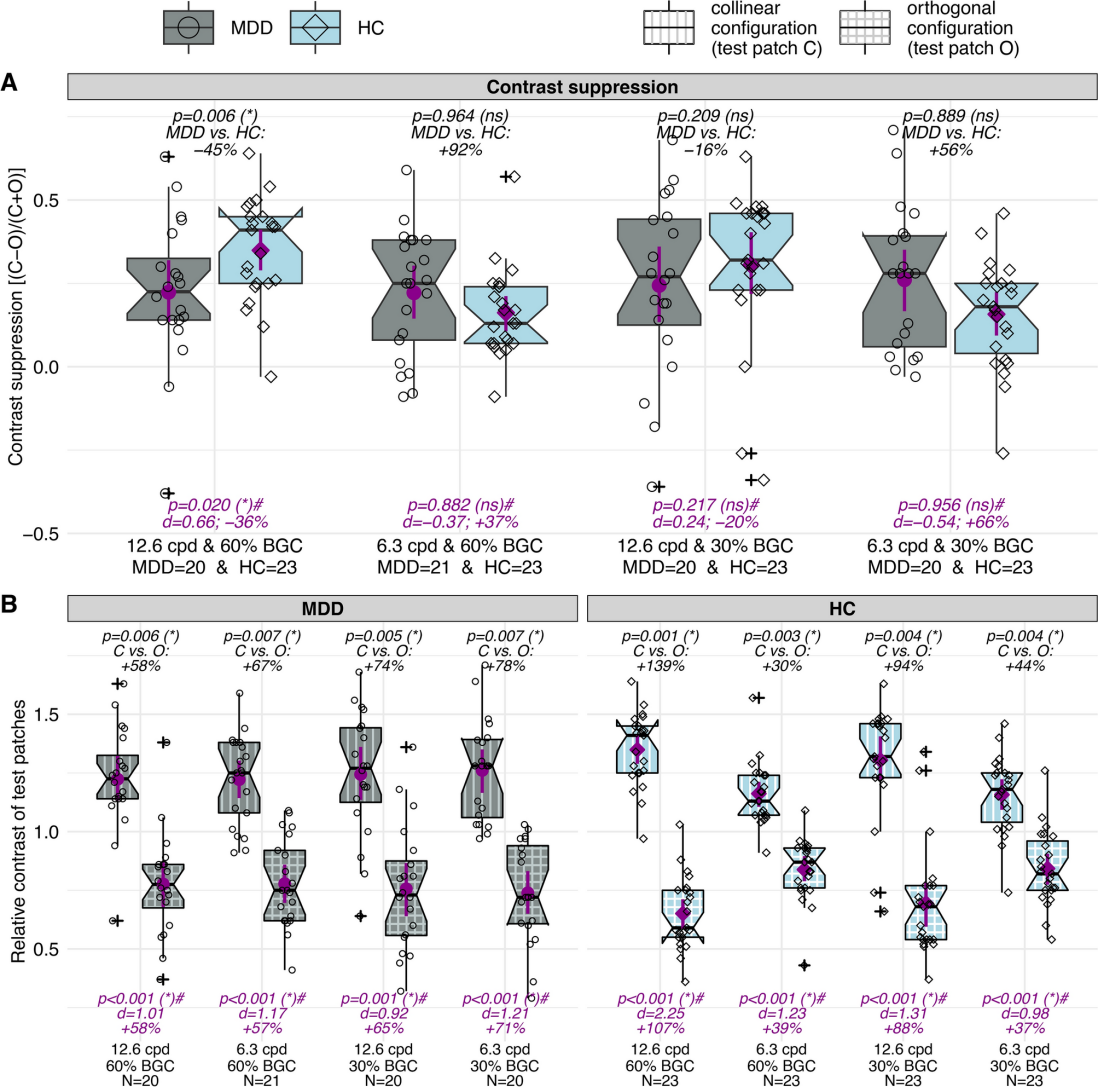
Contrast suppression. **A** Group comparisons of contrast suppression scores ([C−O]/[C+O]; compare Fig. 1) for all conditions. **B** Comparing relative contrasts between collinear and orthogonal test-patches for all conditions and both groups separately. *P*-values and comparisons are based on permutation tests for differences in medians. FDR-adjusted significance levels in brackets. Proportional differences in medians as effect size estimation (A: MDD vs. HC; B: collinear (C) vs. orthogonal (O)). Means and bootstrapped (10,000 replicates) 95% confidence intervals are displayed superimposed on the boxes as magenta-colored points and lines, respectively. *P*-values from parametric statistics (t-tests) and Cohen’s *d* as effect size estimation are annotated below the boxes. Individual data points for MDD (circles) and HC (diamonds) are shown. Abbreviations: BGC = background contrast (%); C = collinear background; *d* = Cohen’s *d*; FDR = false discovery rate; HC = healthy controls; MDD = patients with major depressive disorder; N = number of participants; ns = not significant; O = orthogonal background; * = significant; # = not adjusted.

In both low spatial frequency (6.3 cpd) conditions, contrast suppression in HC showed attenuation compared with MDD patients. However, these differences were not statistically significant (6.3 cpd and BGC 60%: *p* = 0.964, MDD vs. HC: +92%; 6.3 cpd and BGC 30%: *p* = 0.889, MDD vs. HC: +56%) (Fig. 2A; Supplementary Table 1A).

Moreover, a Bland–Altman analysis comparing repetitions of measures, revealed diametrically opposed “biases” in the MDD group for the low background contrast conditions (BGC 30%). While for the fine grating (12.6 cpd) contrast suppression scores in MDD were on average higher in the second replicate of the measurement, smaller average contrast suppression scores were detected in the second replicate using the low spatial frequency grating (6.3 cpd) (Supplementary Fig. 1).

A subsequent within group analysis revealed that contrast suppression in the HC group was significantly reduced for the coarse compared to the fine grating (BGC 60%: *p* = 0.001, 6.3 vs. 12.6 cpd: −68%; BGC 30%: *p* = 0.012, 6.3 vs. 12.6 cpd: −44%), while contrast suppression scores in MDD did not change with the spatial properties of the stimulus (BGC 60%: *p* = 0.803, 6.3 vs. 12.6 cpd: +11%; BGC 30%: *p* = 0.822, 6.3 vs. 12.6 cpd: +4%).

By comparing the relative contrasts of both test patches (collinear vs. orthogonal configuration) for both groups and all conditions individually, we confirmed that contrast suppression (higher relative contrast of test patches from the collinear configurations) was evident in all tested conditions and in both groups (Fig. 2B; Supplementary Table 1B).

### Correlation analysis with depressive symptoms and visual acuity

**Fig. 3:**
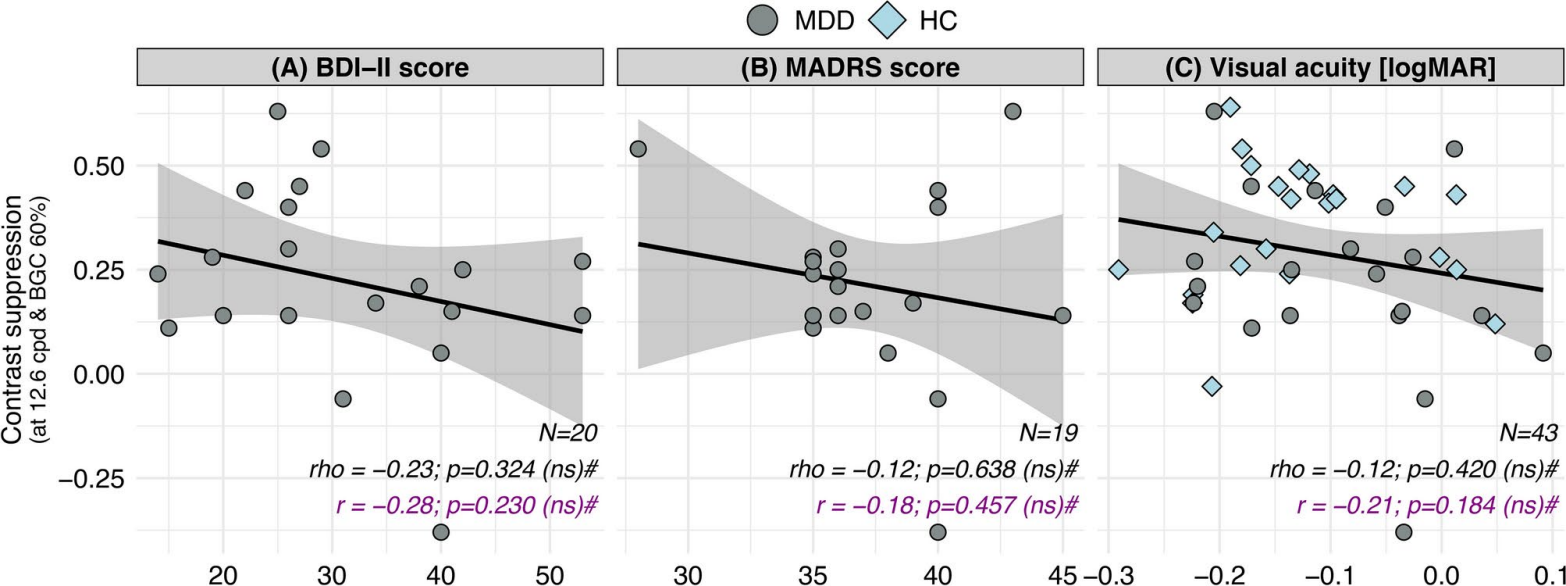
Correlation analysis. Contrast suppression scores of the condition using high contrast (BGC 60%) and spatial frequency (12.6 cpd) for the gratings in relation to depressive symptoms of patients with MDD according to the BDI-II (**A**) or the MADRS scores (**B**) and in relation to the visual acuity in logMAR of all participants (**C**). Spearman’s *rho* was calculated and statistically evaluated using permutation tests (10,000 replicates). Below (magenta-colored) Pearson’s correlation coefficient *r* and corresponding *p*-values are shown for parametric testing. Abbreviations: BDI-II = Beck Depression Inventory II^18^; BGC = background contrast (%); HC = healthy controls; MADRS = Montgomery-Åsberg Depression Rating Scale ^17^; MDD = patients with major depressive disorder; N = number of participants; ns = not significant; *r* = Pearson’s correlation coefficient; *rho* = Spearman’s correlation coefficient; # = no FDR adjustment.

Contrast suppression scores of the high contrast (BGC 60%) and high spatial frequency (12.6 cpd) condition showed no significant correlation with the BDI-II or MADRS scores in the MDD group (Fig. 3A and B) or the visual acuity (logMAR) of all participants (Fig. 3C).

### Descriptive subgroup analysis for medicated and unmedicated MDD

Contrast suppression scores from the fine grating (12.6 cpd) and high background contrast (60%) condition were further evaluated by descriptively comparing medicated (N = 13) and unmedicated (N = 7) patients with MDD to the HCs (N = 23) (Supplementary Fig. 2).

In comparison to HC, unmedicated MDD patients exhibited a larger reduction in contrast suppression scores (−59% vs. HC) than medicated MDD patients (−24% vs. HC) (Supplementary Fig. 2).

## Discussion

We investigated contrast suppression in 21 individuals with a MDD and 23 HC applying a psychophysical examination. Participants were tasked with equally adjusting the perceived contrasts between two vertical central patches, one embedded in a horizontally oriented background grid (orthogonal configuration), the other in a vertically oriented (collinear configuration).

Center-surround contrast suppression is specific to orientation and preferably occurs when the central test grating and the surround grating have the same direction (collinear configuration)^35^, resulting in a reduction of the perceived contrast of the inner grid. We tested four different stimulus conditions using two spatial frequencies (6.3 cpd and 12.6 cpd) and two grating contrasts (60% and 30% background contrast).

A significantly reduced contrast suppression in MDD compared to HC was found in the condition with the high background contrast of 60% and the finer grating with a spatial frequency of 12.6 cpd (*p* = 0.006, MDD vs. HC =  −45%).

Our finding of reduced contrast suppression in patients with MDD is in line with the results of Salmela et al.^3^. However, while we focused on patients with unipolar MDD, Salmela et al.^3^ additionally investigated patients with bipolar or borderline personality disorder and baseline major depressive episodes and showed similarly reduced contrast suppression in these collectives. A lower retinal contrast gain in MDD^1,36^ and a reduced cortical feedback are discussed as possible underlying factors to explain reduced contrast suppression in MDD^3^.

As dopaminergic or serotonergic neurotransmission can affect ERG responses^37^, the reported reduced PERG retinal contrast gain in unmedicated and medicated MDD patients^1^ may reflect alterations in the dopaminergic neurotransmission in MDD^38^, which likely affects subsequent information processing and is probably associated with a reduced contrast sensitivity observed in MDD^50^. Salmela et al.^3^ reported lower contrast suppression which was not associated with age, sex or antidepressant medication.

Some studies report that suppression effects in behavioral data can be attributed to surround suppression effects on the neuronal level, rising from the classical receptive field (center-surround) organization found at various levels along the visual pathways^35^. It is thought that both feedforward (retinal and lateral geniculate nucleus level) and feedback (cortical level) circuits might contribute to neuronal and perceptual contrast suppression effects^3,35^.

Reduced concentrations of the inhibitory neurotransmitter GABA^39^ have been detected in the visual occipital cortex in MDD supporting the hypothesis of diminished visual inhibition^40^ which might contribute to reduced contrast suppression in MDD.

Studies report that the size of the receptive fields, surround suppression and response amplitude are modulated by cortical feedback^41^. Surround suppression refers to the phenomenon where orientation-specific responses to stimuli in the center of the receptive field are suppressed by similarly oriented stimuli in the surrounding area of the receptive field^42^. The question of whether cortical circuits play a role in surround suppression or if the phenomenon is exclusively transmitted from earlier stages of visual processing is not yet resolved^43^. However, GABA interneurons co-mediate center-surround suppression^44^ further supporting the hypothesis that reduced feedback inhibition may contribute to diminished contrast suppression in MDD.

While contrast suppression effects were subdued in HC when presenting coarser gratings with a spatial frequency of 6.3 cpd, MDD patients adjusted the contrasts of the test patches regardless of the spatial frequency of the gratings.

It was reported that retinal and cortical adaptation effects seemed to be specific to high spatial frequencies^45^. Adaptation mechanisms are thought to help adjust the processing of information to the current sensory input by modulating tuning curves towards the adaptor^46^ and even can increase the effects of contrast suppression^47^. Therefore, it might be conceivable that MDD patients lack proper adaptation especially during viewing gratings with high spatial frequencies resulting in a loss of contrast suppression, probably explaining the in-sensitivity to spatial frequencies in the current experiment. Diminished retinal PERG responses to fine checkerboard stimuli^48^ alongside reduced PERG contrast gain^1^ support this hypothesis and reinforce a retinal origin contributing to perceptual contrast suppression alterations in MDD. Nevertheless, future studies are required to investigate the underlying causes of the lack of spatial frequency specificity on contrast suppression in MDD patients.

### Limitations

Future studies need to investigate the replicability of reduced contrast suppression in MDD in a larger sample and evaluate the effects of stimulus parameters such as spatial frequency, in more detail.^3^

We examined patients who received antidepressant medication for a maximum of 14 days and were assessed and diagnosed by an experienced specialist in psychiatry and psychotherapy. Thus, only patients with a severe depressive episode according to the ICD-10 criteria, who were not in remission, were included. An impact of the antidepressant medication cannot be ruled out. However, Salmela et al.^3^ found no association of contrast suppression and the intake of antidepressant medication. Furthermore, our descriptive subgroup analysis revealed that contrast suppression, in comparison to HC, was more attenuated in unmedicated, than in the medicated MDD patients. This observation makes it less likely that antidepressive medication was responsible for the reduction in contrast suppression in MDD.

Moreover, we measured contrast suppression with a subjective behavioral test. Depression is associated with difficulties in attentional processing and reduced attention span which might have an impact on our results of reduced contrast suppression in MDD, which was probably reflected in the higher number of outliers in the MDD data sets and the discrepancy between repeated measures in the “more difficult” low background contrast conditions (Supplementary Fig. 1).

Regarding the absence of a significant correlation between the severity of depressive symptoms and contrast suppression scores, it is important to note that correlation coefficients are prone to range restriction^49^. Insufficient data range and limited dispersion can significantly reduce the sensitivity of such coefficients^30,49^. For this reason, we opted for a Bland–Altman analysis^29^ (Supplementary Fig. 1) to evaluate the reliability of repeated measures. The biases observed between the first and second repetitions in both the MDD and HC groups may indicate adaptation processes. Future studies should investigate such possible effects on contrast suppression in more detail and should additionally include participants with mild to moderate depressive symptoms to enable a more comprehensive correlation analysis.

Moreover, we did not record or limit the response times of participants and therefore cannot draw conclusions about how long the stimuli were viewed. In order to exclude that the orientation-dependent differences at the border between inner and outer grating modulated the perceptual effect, we conducted a control experiment with 10 HC as detailed in Supplementary Fig. 3. This confirmed that the border configuration had not affected contrast suppression (Supplementary Fig. 3).

### Summary

We examined 21 MDD patients and 23 HC using a contrast suppression test. Patients with MDD exhibited reduced contrast suppression compared to HC when backgrounds and test patches were a fine grid pattern with higher overall contrast. This supports previous studies demonstrating diminished retinal (PERG) contrast gain in MDD patients and supports the hypothesis of an altered dopaminergic neurotransmission in MDD which probably also is linked to alterations in cortical processes.

## Supplementary Information

Supplementary Information 1.

## Data Availability

Data as well as the R code for statistical analysis, is available from the corresponding author and EF on request.
