# Supplementary material for: Alterations in center-surround contrast suppression in patients with major depressive disorder
Source: Sci Rep. 2024 Nov 15;14:28160. doi: 10.1038/s41598-024-78584-z (PMC11568198; doi:10.1038/s41598-024-78584-z)
Supplement: Supplementary file 1 — Supplementary Information 1. [file 41598_2024_78584_MOESM1_ESM.docx]

**Supplementary material to: Alterations in center-surround contrast suppression in
patients with major depressive disorder**

**Table of contents**

- Page 2: Supplementary Figure 1: Bland-Altman Analysis with mean absolute trial differences (MATD) as estimates for reliability.
- Page 3: Supplementary Table 1: Median-based robust analyses with *p*-values from permutation tests comparing differences in medians and additional *p*-values and Cohen’s *d* from parametric analyses (t-tests)
  1. Group comparisons (MDD vs. HC) of contrast suppression scores
  2. Within group comparisons of relative contrasts of test patches (collinear vs. orthogonal background)
- Page 4: Supplementary Figure 2: Descriptive subgroup analysis for contrast suppression scores from the fine grating (12.6 cpd) and the high background contrast (60%) condition comparing medicated and unmedicated MDD to HC
- Page 5: Supplementary Figure 3: Additional experiment on 10 HC investigating possible effects of additional edge information in the orthogonal stimulus configuration on contrast suppression.

**References**

1. Altman, D. G. & Bland, J. M. Measurement in Medicine: The Analysis of Method Comparison Studies. *The Statistician* 32, 307 (1983).

2. Bland, J. M. & Altman, D. G. Statistical methods for assessing agreement between two methods of clinical measurement. *Lancet* 1, 307–310 (1986).

3. Bunce, C. Correlation, Agreement, and Bland–Altman Analysis: Statistical Analysis of Method Comparison Studies. *American Journal of Ophthalmology* 148, 4–6 (2009).

**
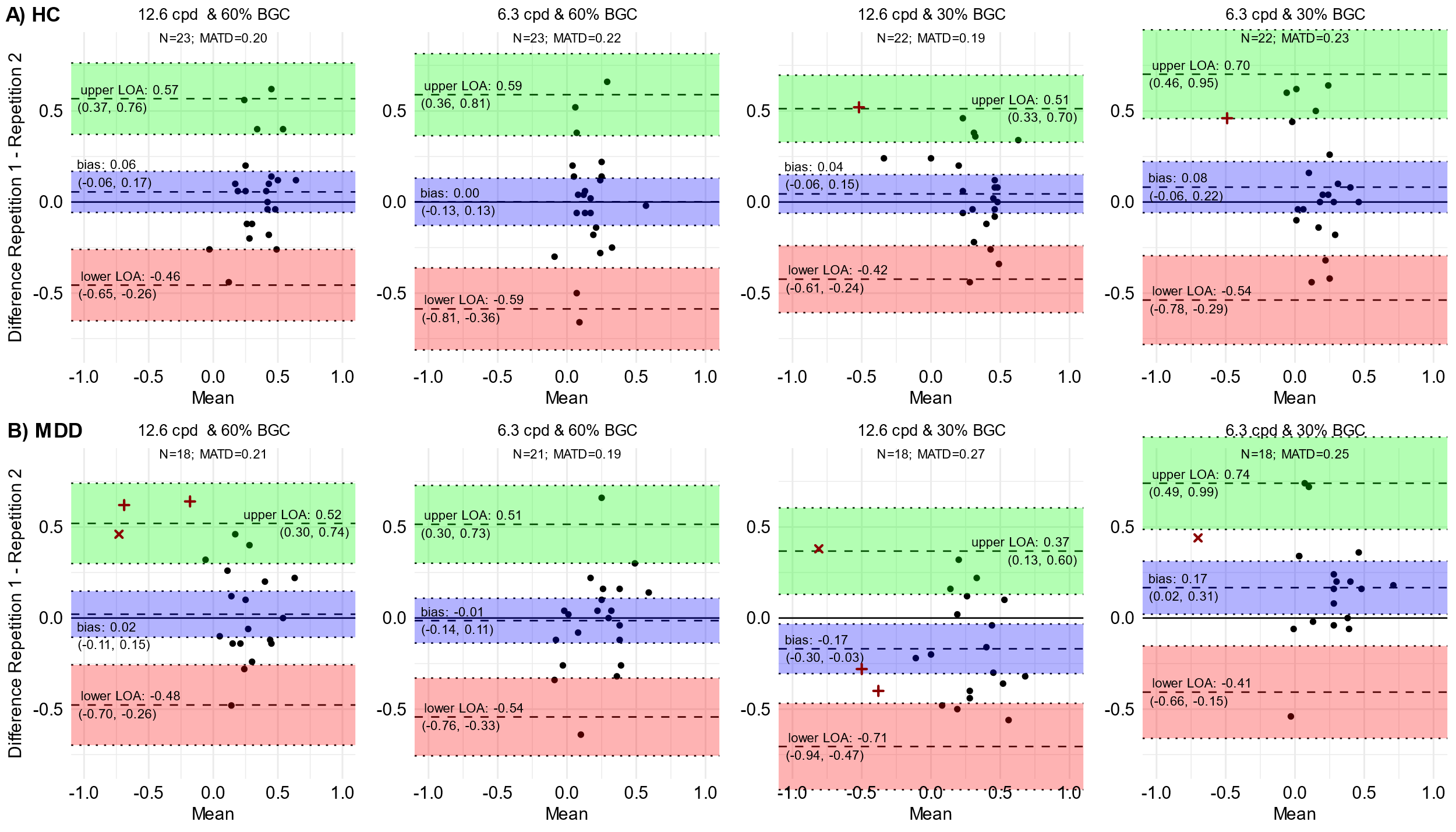
**

**Supplementary Figure 1: Bland-Altman Analyses for both groups and all conditions.**

Test-retest reliability between repetitions of measures was assessed using the limits of agreement (LOA) method suggested by Bland and Altman ^1–3^. Participants providing data from both replicates were included in the analysis (N). Participants showing outliers were not part of the analysis but are additionally marked as “plus” (+) signs when one of two repetitions was detected as outlier, or as “multiplication” sign (×) when both replicates were outliers. The “bias” (average agreement) corresponds to the mean difference between replicates the upper and lower limits of agreement (LOA) to the mean ± 1.96 × standard deviation of the difference. 95% confidence intervals for the bias and both LOAs are additionally depicted. The mean absolute trial differences (MATD) are annotated on top of each plot as estimates for reliability.

**Supplementary Table 1: A:** Group comparisons (MDD vs. HC) of contrast suppression scores for all conditions assuming lower contrast suppression in MDD (unpaired one-sided tests). **B:** Results from the (two-sided) within group comparisons for relative contrasts of test patches (collinear vs. orthogonal backgrounds). Numerical data are summarized by the medians and 95% bootstrapped (10,000 replicates) confidence intervals (CI) and the number of counts (N). *P*-values for the differences in medians were computed by permutation tests (10,000 replicates) (robust statistic). FDR-adjusted significance levels in brackets. For comparability reasons we additionally provide *p*-values (unadjusted significance levels; #) from t-tests (parametric statistics). As effect size estimations the proportional differences of MDD compared to HC medians (robust) or means together with Cohen’s *d* (parametric statistics) are depicted (MDD vs. HC).

| **A:** Group comparisons (MDD vs. HC) of contrast suppression scores | | | | | | |
| --- | --- | --- | --- | --- | --- | --- |
| BGC [%]/ SF [cpd] | **MDD** | | **HC** | | *p*-values (robust/ parametric) | MDD vs. HC: medians/ Cohen’s d (means) |
|  | N | 95% CI | N | 95% CI |  |  |
| **60/12.6** | **20** | **0.22 [0.14, 0.29]** | **23** | **0.41 [0.25, 0.45]** | **0.006 (*)/ 0.020 (*)#** | **−45%/ *d*=0.66 (−36%)** |
| 60/6.3 | 21 | 0.25 [0.10, 0.36] | 23 | 0.13 [0.08, 0.21] | 0.964 (ns)/ 0.882 (ns)# | +92%/ *d*=**−**0.37 (+37%) |
| 30/12.6 | 20 | 0.27 [0.16, 0.42] | 23 | 0.32 [0.28, 0.46] | 0.209 (ns)/ 0.217 (ns)# | −16%/ *d*=0.24 (−20%) |
| 30/6.3 | 20 | 0.28 [0.08, 0.38] | 23 | 0.18 [0.06, 0.25] | 0.889 (ns)/ 0.956 (ns)# | +56%/ *d*=**−**0.54 (+66%) |
| **B:** Within group comparisons of relative contrasts of test patches (collinear vs. orthogonal background) | | | | | | |
| BGC [%]/ SF [cpd] | **MDD** 95% CI | | **HC** 95% CI | | Background orientation | |
| 60/12.6 | 0.78 [0.71, 0.86] | | 0.59 [0.55, 0.75] | | Orthogonal | |
|  | 1.23 [1.14, 1.30] | | 1.41 [1.26, 1.43] | | Collinear | |
|  | 0.006 (*) <0.001 (*)# (*d*=1.01) | | 0.001 (*) <0.001 (*)# (*d*=2.25) | | *p*-value (robust) *p*-value (parametric) (Cohen’s *d*) | |
| 60/6.3 | 0.75 [0.64, 0.90] | | 0.87 [0.79, 0.93] | | Orthogonal | |
|  | 1.25 [1.10, 1.36] | | 1.13 [1.08, 1.24] | | Collinear | |
|  | 0.007 (*) <0.001 (*)# (*d*=1.17) | | 0.003 (*) <0.001 (*)# (*d*=1.23) | | *p*-value (robust) *p*-value (parametric) (Cohen’s *d*) | |
| 30/12.6 | 0.73 [0.58, 0.84] | | 0.68 [0.54, 0.72] | | Orthogonal | |
|  | 1.27 [1.16, 1.42] | | 1.32 [1.23, 1.46] | | Collinear | |
|  | 0.005 (*) 0.001 (*)# (*d*=0.912) | | 0.004 (*) <0.001 (*)# (*d*=1.31) | | *p*-value (robust) *p*-value (parametric) (Cohen’s *d*) | |
| 30/6.3 | 0.72 [0.61, 0.92] | | 0.82 [0.76, 0.90] | | Orthogonal | |
|  | 1.28 [1.08, 1.38] | | 1.18 [1.10, 1.24] | | Collinear | |
|  | 0.007 (*) <0.001 (*)# (*d*=1.21) | | 0.004 (*) <0.001 (*)# (*d*=0.98) | | *p*-value (robust) *p*-value (parametric) (Cohen’s *d*) | |

Abbreviations: BGC = background contrast (%); CI = 95% confidence interval; FDR = false discovery rate; HC = healthy controls; MDD = patients with major depressive disorder; N = number of participants; ns = not significant SF = spatial frequency (cpd); * = significant; # = not adjusted.


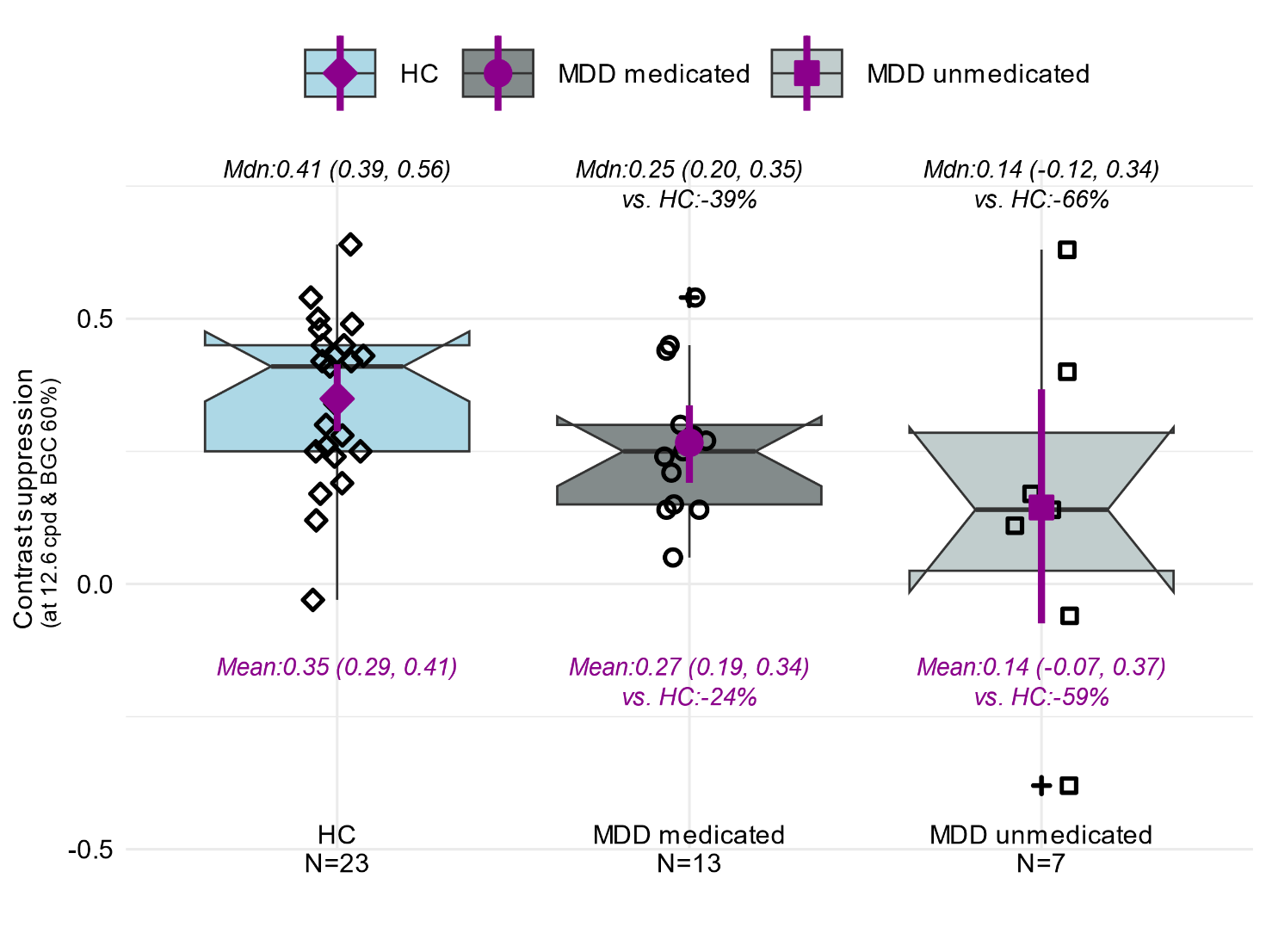


**Supplementary Figure 2:** Descriptive subgroup analysis for contrast suppression scores from the fine grating (12.6 cpd) and high background (60%) condition comparing medicated (N=13) and unmedicated (N=7) MDD to HC (N=23). Magenta-colored points and point ranges superimposed on the boxes show means and corresponding 95% confidence intervals (CI) (bootstrapped; 10,000 replicates). Text annotations: above the boxes medians (Mdn) and 95% CIs, below the boxes means and 95% Cis (magenta-colored). The proportional deviation of MDD medians or means from HC medians or means is additionally depicted as % (“vs. HC”).

Abbreviations: BGC = background contrast (%); CI = 95% confidence interval; cpd = cycles per degree; HC = healthy controls; N = number of participants; MDD = patients with major depressive disorder; Mdn = median.

|  |
| --- |
|  |
| 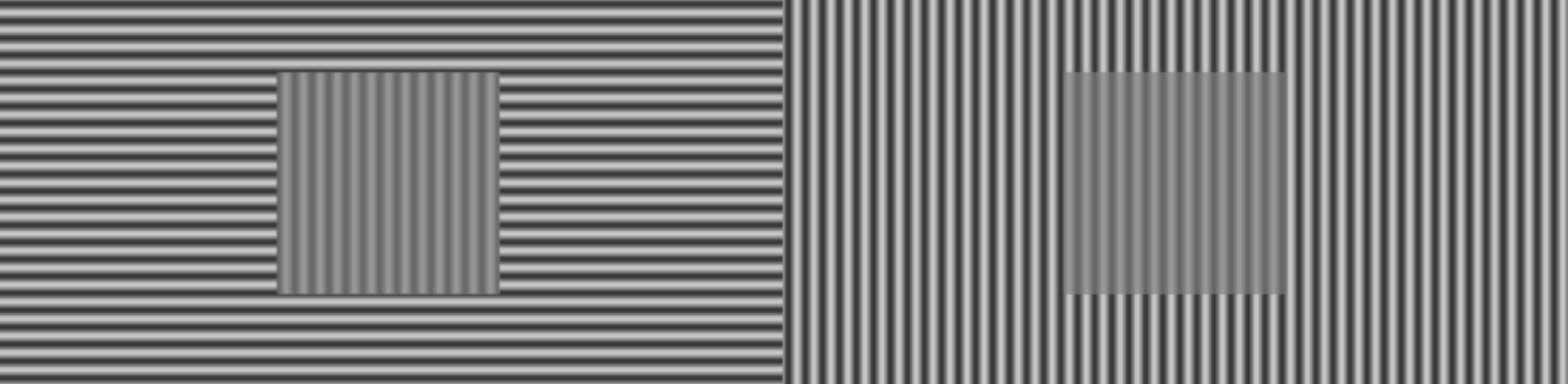  **A** |
| 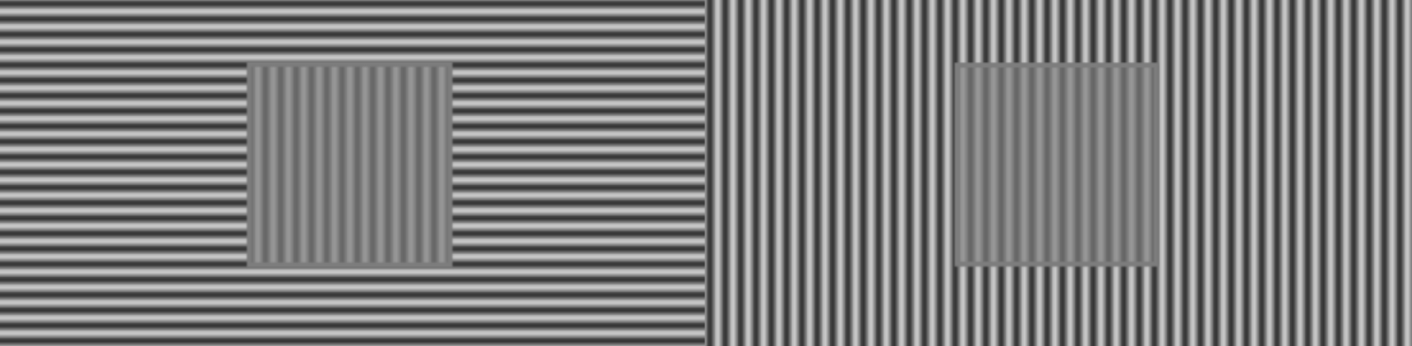  **B** |
| 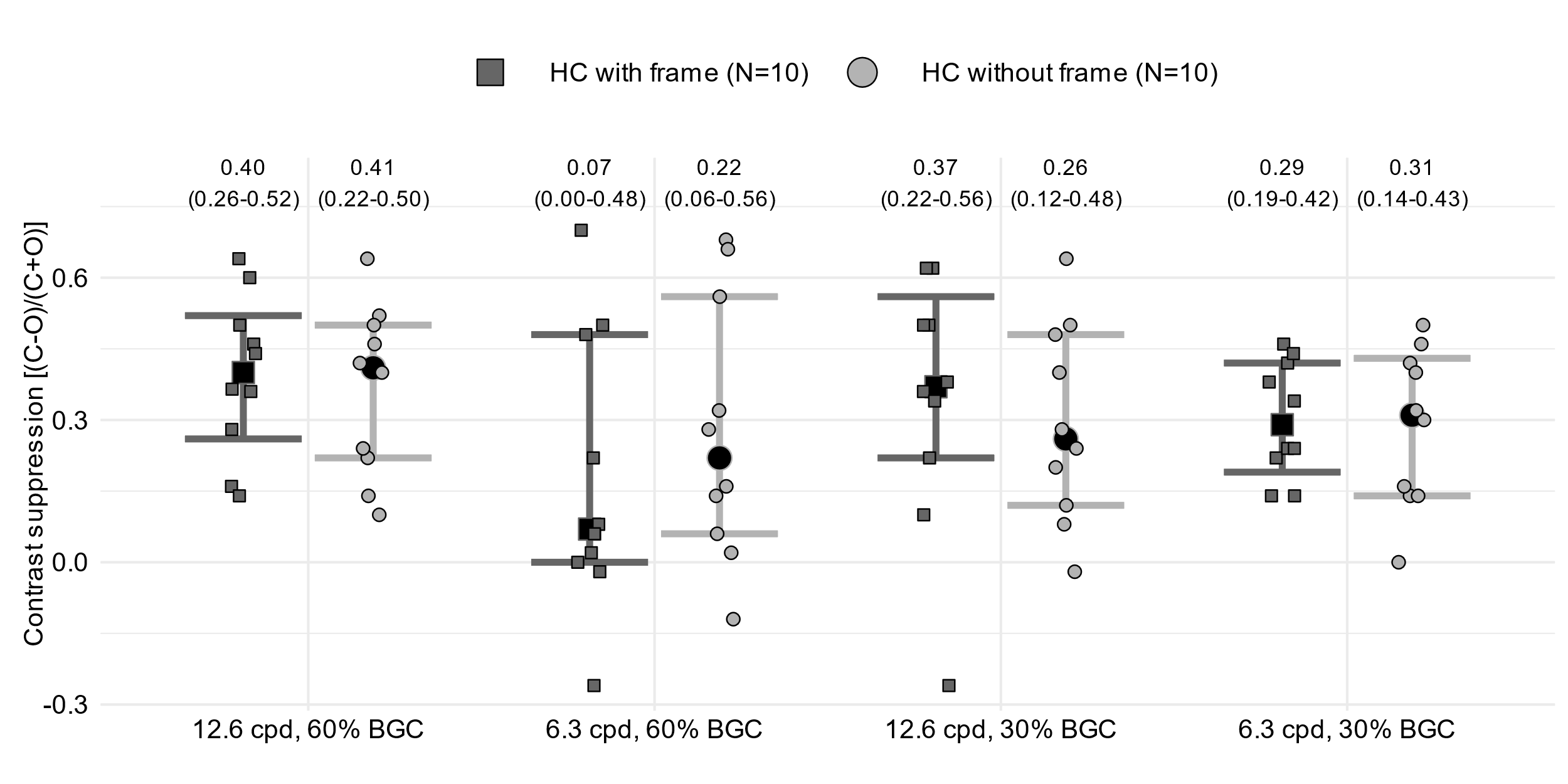  **C** |

**Supplementary Figure 3:** Additional experiment on 10 HC investigating possible effects of additional edge information in the orthogonal stimulus configuration on contrast suppression. To evaluate if the perceivable edges between inner and outer grating in the orthogonal stimulus configuration had influenced contrast suppression in the main experiment, we conducted a control experiment where we hid the border between gratings by superimposing a medium grey rectangle, thereby equalizing edge effects between the orthogonal and the collinear configuration. **A** upper row: Enlarged representation of the original condition (12.6 cpd & 60% background contrast (BGC)) with perceivable edges in the orthogonal configuration. **B** upper row: The same condition with superimposed rectangles. All subjects completed the original condition without the superimposed frame and the control condition with hidden edges. **C**: Data of the control experiment for both tests (with frame and without frame) and all stimulus conditions. The medians (black dots) and 95% bootstrapped (10,000 replicates) confidence intervals (CI) (error bars) are depicted and annotated on top of the plot. Individual data points are superimposed on the plot. Results show that contrast suppression scores are similar for both tests, suggesting that contrast suppression in the main experiment was not confounded by edge effects in the orthogonal configuration.
